# Fertilizer regime impacts on abundance and diversity of soil fauna across a poplar plantation chronosequence in coastal Eastern China

**DOI:** 10.1038/srep20816

**Published:** 2016-02-09

**Authors:** Shaojun Wang, Han Y. H. Chen, Yan Tan, Huan Fan, Honghua Ruan

**Affiliations:** 1 Department of Environmental Science and Engineering, Southwest Forestry University, Bailongshi, Kunming 650224, P.R. China; 2 Co-Innovation Center for Sustainable Forestry in Southern China, College of Biology and the Environment, Nanjing Forestry University, Nanjing 210037, P.R. China; 3 Faculty of Natural Resources Management, Lakehead University, 955 Oliver Road, Thunder Bay, ON, Canada P7B 5E1

## Abstract

Soil fauna are critical for ecosystem function and sensitive to the changes of soil fertility. The effects of fertilization on soil fauna communities, however, remain poorly understood. We examined the effects of fertilization form and quantity on the abundance, diversity and composition of soil fauna across an age-sequence of poplar plantations (i.e., 4-, 9- and 20-yr-old) in the coastal region of eastern China. We found that the effects of fertilization on faunal abundance, diversity, and composition differed among stand ages. Organic fertilizers increased the total abundance of soil fauna, whereas low level inorganic fertilizers imparted increases only in the 4- and 9-yr-old stands. The number of faunal groups did not change with fertilization, but Shannon’s and Margalef diversity indices increased under low level organic fertilization, and decreased under inorganic fertilization in the 9- and 20-yr-old stands. Community composition of soil fauna differed strongly with fertilization and stand age. The changes in soil fauna were strongly associated with the changes in microbial biomass carbon, dissolved organic carbon and nitrogen, and available phosphorus and potassium. Our findings suggest that the responses of soil fauna to fertilization may be mediated through the fertilization effects on soil nutrient availability.

Soil faunal communities are critical for ecosystem functionality, with respect to direct and indirect interactions with plants, nutrient, and organic matter cycling^1^,^2^,^3^,^4^. The composition and diversity of soil fauna are typically explained by the top-down effects of predators and the bottom-up effects of resource availability^5^,^6^,^7^. Soil fauna have a strong association with soil nutrient availability^8^ and are sensitive to changes in the root and soil physicochemical environments, which may be affected by organic and inorganic fertilizers^9^,^10^,^11^. These findings suggest that soil fauna comprise a viable and valuable indicator of soil fertility, and the indices or taxa of soil fauna can be used for indicating changes in soil fertility and offer a promising means for scientists to evaluate the effectiveness of soil management.

Poplar trees are widely cultivated throughout much of the world, as well as in China due to their rapid-growth, short-rotation period, capacity for enhancing wood supplies, and high economic value^12^,^13^. Particularly, poplar plantations may contribute to a great extent toward the alleviation of global climate change because of their high biomass generation and carbon sequestration capabilities^14^,^15^. The maintenance of soil fertility is of paramount importance to meet the nutrient demands of high production for plantation cultivation, as well as contributing to the mitigation of the effects of global climate change^16^,^17^. Fertilization has a strong impact on the soil-plant interface through the rhizosphere boundary, which subsequently impacts the productivity and sustainability of plantations^18^. Organic and inorganic fertilizers may have beneficial, neutral, or harmful effects on the interactions among plants, soil biota, and the soil environment^9^. Previous studies have focused primarily on how fertilizers influence plant production and soil chemistry^19^,^20^. Few studies have, however, examined the effects of fertilizers on soil fauna in managed tree plantations.

Changes in the soil faunal community under the influence of fertilization may be determined primarily by the quantity and quality of food (i.e., a bottom-up approach), and modification of soil physical and chemical properties^21^,^22^. The application of both organic and inorganic fertilizers to ecosystems has been shown to increase the populations and diversity of soil fauna (e.g., microarthropods and nematode)^23^,^24^. The increase in soil faunal population and diversity occurs in response to increased substrate availability, aboveground biomass yield, and altered soil physicochemical conditions under fertilizer applications^9^,^25^,^26^. Inorganic fertilization provides readily available nutrients for plant growth, which may bring about higher food sources for soil faunal populations and microbial activity through the input of plant and root residues^23^,^27^. Organic fertilization improves soil macro- and micro-aggregation, and results in a resource-mediated increase in soil faunal populations^24^. The negative effects of fertilizer application, however, have also been reported, specifically the effects of inorganic fertilizers. The abundance and diversity of soil microarthropods have been reported to decline following the application of nitrogenous fertilizer to soil^28^,^29^. Therefore, there remains much uncertainty as to the impacts of fertilizer application on the abundance, diversity, and communities of soil fauna.

The relationship between fertilization and soil fauna has received increased attention; however, research pertaining to the effects of fertilizer regimes on the saline-alkali soils of coastal areas is lacking. Saline-alkali soils with an area of approximately 3.69 × 10^7^ hm^2^ are extensively distributed in the north and coastal regions of China. Saline-alkali soils are low in organic matter and nitrogen. Fertilization comprises an essential technique for maintaining and improving plant yields in saline-alkali soils. Because plantations of varying stand age differ in soil fertility demands, fertilization may lead to various changes in soil properties, and in soil fauna communities with stand age. Fertilization may lead to rapid changes in soil fertility, which could be determined by microbial biomass carbon, dissolved organic carbon, and other nutrients, to exert an effect on soil biology^30^,^31^. We undertook a 14-month experiment to evaluate the effects of inorganic (NPK compound fertilizer) and organic (soybean cake) fertilizer on soil faunal communities across three stand ages of poplar plantations on saline-alkali soils in the coastal regions of China. The chief queries of this study were: (1) What are the effects of organic and inorganic fertilization on the abundance, composition and diversity of soil fauna across different aged stands, and (2) How are the changes in soil faunal communities associated with the changes of soil properties under fertilization?

## Results

### Total abundance (TA) of soil fauna community

**Figure 1:**
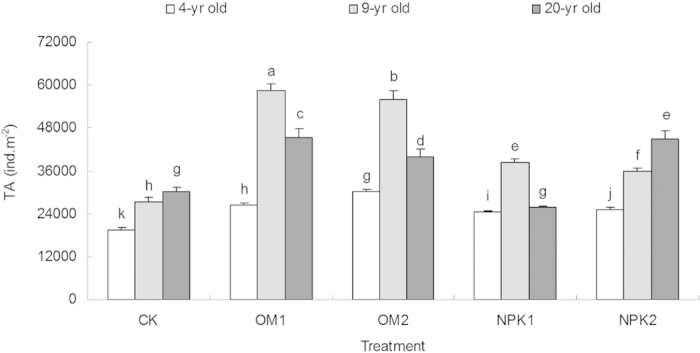
Effects of stand age and fertilization on the total abundance (TA) of soil fauna in four samples, collected from September 2012 to June 2013 across the three age classes (bars indicate SE; n = 3). CK: unfertilized control; OM1: low level of organic matter fertilizer; OM2: high level of organic matter fertilizer; NPK1: low level of inorganic fertilizer; NPK2: high level of inorganic fertilizer. Treatments of TA with the same letter were not significantly different (ANOVA with Tukey-HSD, *p* < 0.05).

The influence of fertilizer treatments on TA varied significantly across the three stand age groups (Table 1, Fig. 1). Organic fertilization at both doses significantly influenced the TA (F = 6.503, *P* < 0.05) in all three age classes, while the low level inorganic fertilization had no significant effect on TA in the 20-yr-old stands. The influence of organic fertilization on the TA was significantly greater than that of inorganic fertilization, where the highest average value (58,386 ind. m^−2^) was recorded for the low level organic fertilization in the 9-yr-old stands (Fig. 1). The increase of TA in response to organic fertilizer application was consistent across all three stand age classes, in comparison with the CK treatment, but with a less magnitude in the 20-yr-old stands (Fig. 1). Except for the inorganic treatment in the 9-yr-old stand, higher TA was observed for samples with the high level inorganic fertilizer. On average, TA was higher for the organic fertilization than for the inorganic fertilization.

### Diversity of soil fauna

**Figure 2:**
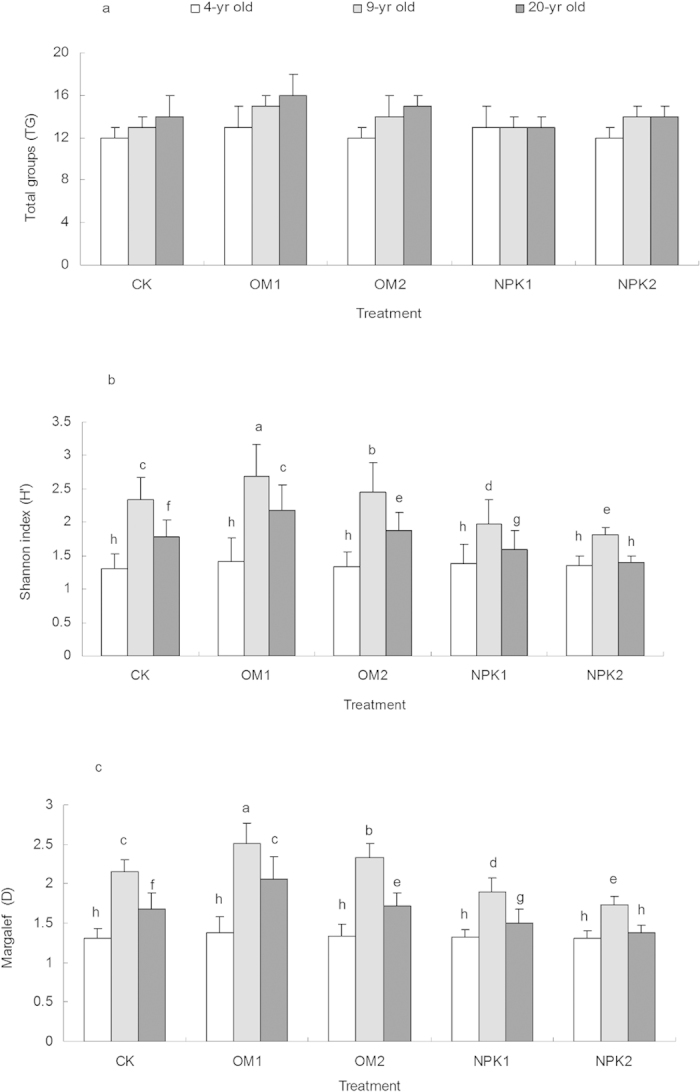
Effects of stand age and fertilization on faunal diversity. (**a**) Total groups (TG); (**b**) Shannon-Wiener index (H′); (**c**) Margalef index (D) across the three age classes (bars indicate SE; *n* = 3). CK: unfertilized control; OM1: low level of organic matter fertilizer; OM2: high level of organic matter fertilizer; NPK1: low level of inorganic fertilizer; NPK2: high level of inorganic fertilizer. Treatments with the same letter were not significantly different (ANOVA with Tukey-HSD, *p* < 0.05).

Although there was no difference in total number of faunal groups associated with stand age and treatment (Table 1, Fig. 2), the stand age, fertilizer treatment and their interactions had a significant influence on Shannon-Wiener index (H’) and Margalef richness index (D) (Table 1). There were differential effects of organic and inorganic fertilizations on these indices across the three age classes (Table 1, Fig. 2). H’ and D in the 4-yr-old stands were not significantly affected by the organic or inorganic fertilization. However, there were increases in 14.5% of H’ and 16.2% of D in the 9-yr-old stands, and increases in 23.9% of H’ and 18.9% of D in the 20-yr-old stands, respectively, with the addition of the low level organic fertilization; whereas a significant decrease in diversity (10.1–22.6% of H’) occurred with the application of the inorganic fertilization. On average, low level organic and inorganic fertilization applications resulted in the higher biodiversity of soil fauna, in comparison with high fertilization levels.

### Composition of soil fauna community

**Figure 3:**
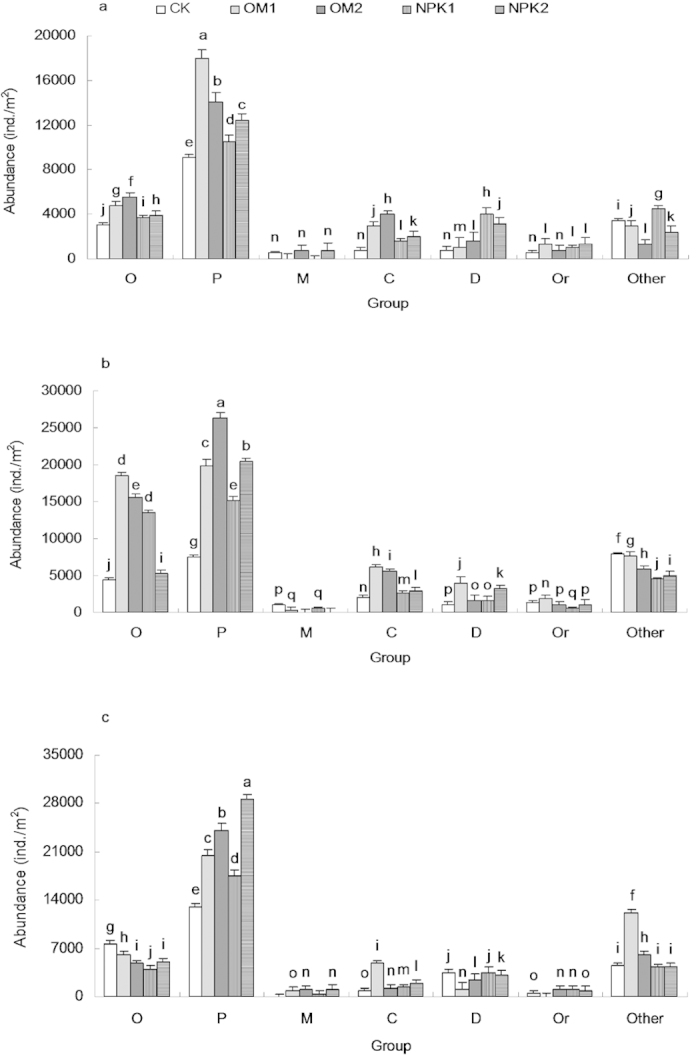
Effects of stand age and fertilization on the abundance in faunal communities across the three age classes (**a**) 4-yr-old; (**b**) 9-yr-old; (**c**) 20-yr-old stands. CK: unfertilized control; OM1: low level of organic matter fertilizer; OM2: high level of organic matter fertilizer; NPK1: low level of inorganic fertilizer; NPK2: high level of inorganic fertilizer. O: Oribatids; P: Prostigmata; M: Mesostigmata; C: Collembola; Di: Diptera larvae; Or: Orthoptera; Other: other soil fauna except for Oribatids, Prostigmata, Mesostigmata, Collembola, Diptera larvae, and Orthoptera (bars indicate SE; n = 3). Treatments of faunal group with the same letter were not significantly different (ANOVA with Tukey-HSD, *p* < 0.05).

Faunal community composition was significantly affected by fertilization regime and age class (Table 2). The effect of the fertilization on the abundance of major soil faunal groups varied among the three age classes (Fig. 3). Prostigmata, Oribatids, Collembola, Mesostigmata, Diptera larvae and Orthoptera comprised the dominant groups, which accounted for more than 80% of the individual proportions at each site. The effects of fertilization, age class, and their interaction on the Prostigmata, Oribatids, Collembola and Diptera larvae were significant (Table 1). Organic and inorganic fertilization significantly increased Prostigmata, Oribatids, Collembola and Diptera larvae in the 4- and 9-yr-old stands (Fig. 3). In the 20-yr-old stands, however, the only significant increases were observed in Prostigmata (F = 7.315, *P* < 0.05) and Collembola (F = 9.428, *P* < 0.05) in response to the organic fertilizer, and Prostigmata (F = 8.022, *P* < 0.05) and Collembola (F = 6.337, *P* < 0.05) in response to the inorganic fertilizer (Fig. 3). Moreover, compared with the CK treatment, the greatest increase in Prostigmata and Oribatid abundance in response to organic and inorganic fertilizations occurred in the 9-yr-old stands, while the lowest increase occurred in the 20-yr-old stands.

### Changes in soil properties and their correlations to soil fauna

The changes in soil properties influenced by fertilizations also varied among the three age classes (Table 3). Organic fertilization significantly increased the microbial biomass carbon (MBC), dissolved organic carbon (DOC), total nitrogen (TN), dissolved organic nitrogen (DON), available phosphorus (AP) and potassium (AK) concentrations over all age classes. The inorganic fertilizer also had a positive effect on the TN, DON, AP and AK in the 4-, 9- and 20-yr-old stands, but the positive effects on DOC were observed with low and high levels of inorganic fertilizations only in the 9-yr-old stands, and the DOC was increased only by high level inorganic fertilization in the 4- and 20-yr-old stands. In the treatments with high fertilizer levels, higher TN, DON, DOC, AP and AK concentrations were usually observed, in comparison with low fertilizer levels.

**Figure 4:**
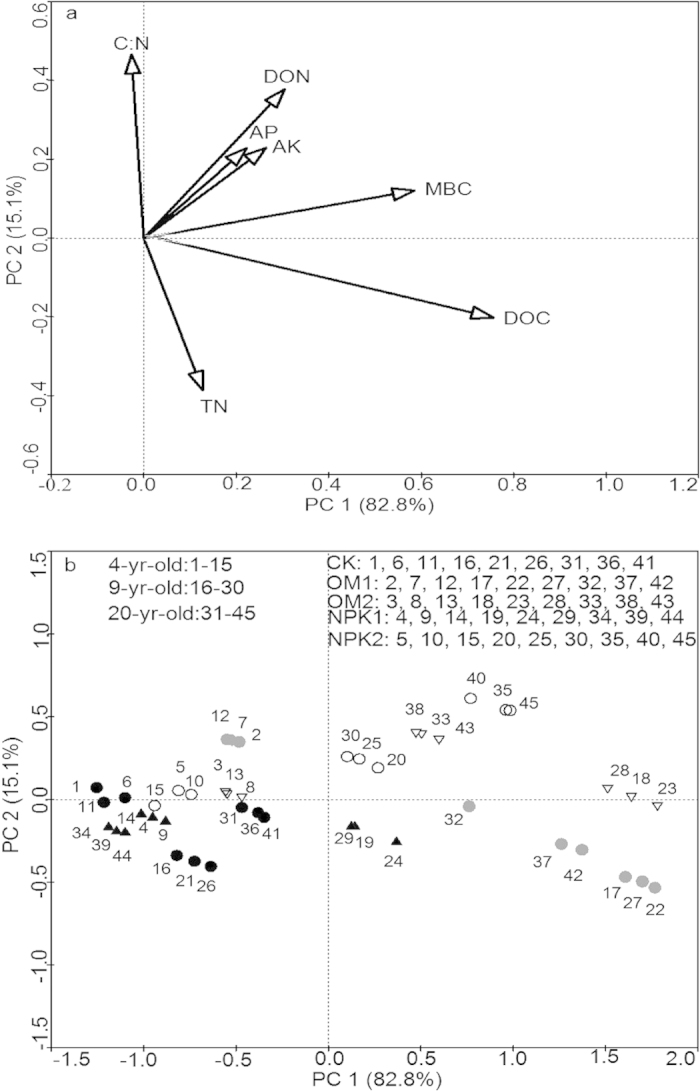
A principal component analysis on the association between soil faunal community composition and soil variables (**a**) loadings of explanatory variables, (**b**) 45 plots labelled by stand age and treatments. CK: unfertilized control; OM1: low level of organic matter fertilizer; OM2: high level of organic matter fertilizer; NPK1: low level of inorganic fertilizer; NPK2: high level of inorganic fertilizer. MBC: microbial biomass carbon; TN: total nitrogen; DOC: dissolved organic carbon; DON: dissolved organic nitrogen; C:N: carbon-nitrogen ratio; AP: available phosphorus; AK: available potassium.

The relationships between soil fauna and soil nutrients varied according to faunal community indices and the abundances of major groups across all sample sites (Table 4). TA was positively correlated to MBC, C:N, DOC, DON, AP and AK, but not to TN. Both H′ and D were positively correlated to MBC, DOC, DON, AP and AK, but not to TN and C:N. Prostigmata were positively correlated with MBC, DOC, TN, DON, AP and AK, and Oribatids, Collembola and Mesostigmata were positively correlated with MBC, DOC, AP and AK. A principal component analysis (PCA) used to examine the changes in faunal community composition as a whole indicated that the first two principal components captured 97.9% of the variation of soil faunal composition, with 82.8% in the first axis, and the second axis explained 15.1% of the variation in faunal composition (Fig. 4a). Soil MBC, AK, AP, DOC, DON had strong positive correlations with PCA axis 1, while soil C:N and TN were correlated with PCA axis 2 positively and negatively, respectively (Fig. 4a). Soil faunal composition differed with stand age and fertilization treatments as stands with different ages and fertilization treatments were well separated in the PCA ordination space (Fig. 4b).

## Discussion

We found that the total abundance of soil fauna was increased by the application of organic fertilizer across three age groups of poplar plantations. Changes in soil faunal communities were primarily determined by food resources^1^,^5^,^21^. Our results add to the previous findings that organic fertilizers have positive effects on soil fauna^24^,^32^,^33^,^34^,^35^. Particularly, Murray *et al.* (2006) demonstrated that an increase in soil faunal abundance was induced by organic fertilization through a direct increase in detritus supply and an indirect modification of soil nutrient environment for fauna^35^,^36^. We found evidence for the increase in the total abundance of soil fauna with organic fertilization through increased soil nutrient availability across all three stand ages of poplar plantations in eastern China.

Inorganic fertilization, on the other hand, had mixed effects on the total abundance of soil fauna. Inorganic fertilization can increase the abundance of soil fauna, but several studies have reported no effect^37^,^38^. The application of inorganic nitrogen results in greater plant biomass production and higher root residue volumes that likely serve as food sources for soil fauna^39^, but the positive effects tend to more apparent in N-limited sites^40^. The saline-alkali soil is known for its deficiency in soil N^41^. In our study, the soil N contents increased from young to old stands. While inorganic fertilizer had on average positive effects on total abundance of soil fauna, the magnitude of the effects was depended on stand age and the quantity of inorganic fertilizer. In particular, no significant effect on soil fauna abundance was observed with the low dose inorganic fertilizer in the soil of the 20-yr-old stands where the N content was the highest among the age classes. It is possible that the soil faunal community is more stable and mature in the old stands, and thus less sensitive to low level inorganic fertilization. Alternatively, high background N contents in the old stands make soil fauna less responsive to low dose inorganic fertilizer.

We found no difference in total number of faunal groups across stand ages and fertilization regimes, nor the Shannon diversity (H’) or Magalef index (D) in the 4-yr-old stands. However, there were significant increases in H’ (14.5–23.9%) and in D (16.2–18.9%) with the application of low level organic fertilizer in the 9- and 20-yr-old stands. Increases in soil faunal diversity occurred largely due to a positive effect of detritus inputs and low-input management on the diversity of the ecosystem^24^. Moderate stress such as in low-input systems, may reduce the likelihood of competitive exclusion, thus allowing other organisms to proliferate. Greater soil fauna diversity is also related to the increased diversity of available food resources of plant roots and litter inputs into soils^42^,^43^. Therefore, low stand productivity, undergrowth plant diversity and fine root input might have been factors that hindered the development of faunal diversity in the 4-yr-old stands.

There was, however, a significant decrease in diversity (10.1–22.6% of H’) in response to the inorganic fertilization in the 9- and 20-yr-old stands. Modifications in management intensity, such as fertilizer application, may alter the soil environment and be expressed as stress. Our observed hump-backed relationship between management disturbance and faunal diversity indicated that severe stress imposed by intensive management could initiate a reduction in soil faunal diversity. In our study, N, P, and K were less deficient in the older stands (Table 3). Therefore, the NPK fertilizer might have acted as an undesired stress that negated increased soil faunal diversity for the old stands.

We found that soil faunal community composition was strongly affected by fertilization and differs with stand age. The abundances of Prostigmata, Oribatids, Collembola and Diptera larvae were increased by organic and inorganic fertilizers in the 4- and 9-yr-old stands. Whereas there was an increase only in Prostigmata and Collembola with organic fertilizer, and an increase in Prostigmata and Collembola with inorganic fertilizer in the 20-yr-old stands. The responses of soil fauna to fertilizers are complex and may be varied according to various specific groups of organisms because of their diversity demands for feeding habits and habitat environments^44^. In consequence, we found that faunal community composition differed strongly with fertilization regimes across our studied age-sequence.

We found that organic and inorganic fertilizations imparted different effects on the soil fertility properties across the three age classes. Previous results from both short- and long-terms fertilization experiments have suggested that significant increases in the DOC, DON, and TN of soils may be typically attained through the introduction of organic amendments, though they can be elevated via inorganic fertilization^30^,^31^,^45^,^46^. The different effects of NPK fertilizer associated with stand age and fertilizer dose are attributable to the fact that different stand ages have different demands for soil nutrients. These diverse effects of organic and inorganic fertilizations on the soil fertility properties may exert differentiated influences on development of soil fauna. Consistent with previous findings^47^, we found that organic fertilizers have a greater positive influence on soil characteristics and root development than inorganic fertilizer.

In the study, increases in the nutrient supplies of soils, resulting from the application of organic and inorganic fertilizations, induce alterations in soil faunal communities. This finding is consistent with previous studies^48^,^49^. We show evidence that MBC, DOC, DON, AP, and AK increased by fertilization, and in turn induced significant changes on the soil faunal community. This might offer a promising chance for scientists to gauge the effectiveness of fertilization on soil fauna. There was a significant correlation between the selected soil properties (e.g., MBC, DOC, TN, DON, AP, and AK), and the indices of soil fauna community (e.g., TA, H′, and D), as well as the abundances of Prostigmata, Oribatids, Collembola, and Mesostigmata across our study plots. These relationships suggest that soil faunal abundance, diversity and composition are sensitive to the changes in soil environment.

## Conclusion

The effects of inorganic and organic fertilizations on the abundance, diversity and composition of faunal community were investigated along an age-sequence of poplar plantations in saline-alkali soils. The influence of fertilization regimes on soil fauna differed among stand age, and fertilization form and quantity. Organic fertilization increased the total abundance of soil fauna in all three stand ages, whereas low level of inorganic fertilization had no significant effect in the 20-yr-old stands. There was a higher diversity of soil faunal community in organic fertilization than in inorganic fertilization. The highest abundance and diversity of soil fauna were generally observed in the low level of organic fertilizer treatments. Soil faunal community composition strongly differed with fertilization regime and stand age. The changes in soil fauna abundance, diversity and composition were associated with the changes of soil properties including microbial biomass carbon, dissolved organic carbon and nitrogen, and available phosphorus and potassium under fertilization. Our results suggest that the changes of soil fauna associated with fertilization regime and stand age are mediated through the changes in soil nutritional environment.

## Methods

### Site description

This study was conducted in the Yellow Sea State Forest Park in the eastern China (32°33′ ~ 57′ N, 102°07′~53′ E). This region is located in the north warm temperate-subtropical transition zone and is under the influence of the monsoon climate. The mean annual temperature in this area is approximately 13.7 °C, with 1051.0 mm of annual rainfall, and an annual average relative humidity of 88.3%. The frost-free period extends for 220 d with an average exposure to sunlight of 2,169.6 hours. This terrain is included in the middle and lower reaches of the alluvial Yangtze River plain, with desalted sandy loam meadow soil.

### Experimental design

There are different stand ages (an area of approximately 3,000 ha) of poplar plantations in the study area, varying from 3- to 23-yr-old. To examine the effects of stand development, three stand ages (4-, 9-, and 20-yr-old) of pure poplar (*Populus deltoides* Marsh) plantations with similar site conditions were sampled and replicated in triplicate with spatial interspersion (at least 500 m apart for the same age stands). The stands along the age chronosequence had the same parent material (basalt), similar altitude (less than 5 m altitude difference), similar initial field management, and similar initial conditions of succession (Table 5). Especially, there were no significant differences of soil properties including pH, soil bulk density and total carbon and nitrogen among the experimental plots.

We employed organic and inorganic fertilizations (each with low and high levels) to examine the effects of fertilization form and quantity on the abundance, diversity and composition of soil fauna across an age-sequence of poplar plantations. In the selected three age classes (4-, 9-, and 20-yr-old) of plantations, fertilizers were applied in June, September and December 2012, and in March 2013, respectively, coinciding with the growing demand of poplar plantations. Within each sample stand, we establish an experimental plot (40 × 50 m), and five subplots of (2 × 2 m) within the plot. Five treatments were randomly assigned to these subplots: (1) unfertilized control (CK), (2) a low level organic matter fertilizer application (OM1, 1875 kg ha^−1^), (3) high level organic matter fertilizer application (OM2, 3750 kg ha^−1^), (4) low level compound fertilizer (NPK1, 750 kg ha^−1^), and (5) high level compound fertilizer (NPK2, 1500 kg ha^−1^). These fertilizer doses have been commonly used in local fertilization practice. The organic fertilizer consisted of soybean cake on a dry weight basis, which was applied throughout the depth of 0–20 cm via trenching. The N:P:K, total organic carbon (TOC), and pH in organic fertilizer were 6:1:2, 326 g kg ^−1^ and 6.45, respectively. The NPK compound fertilizer had a N:P:K ratio of 8:4:3 and pH of 6.92, and was also applied throughout the depth of 0–20 cm^50^.

### Analysis of soil properties and identification of soil fauna

For every subplot, samples from the 0–20 cm soil layer were collected three months subsequent to fertilization (e.g., September and December of 2012, and March and June of 2013). Each of the soil samples consisted of four-five cores (2.5 cm diameter) that were collected for the measurements of soil parameters and microarthropods during each sampling period. Total nitrogen (TN) concentrations in the soil were measured using an elemental analyzer (Elementar, Vario ELIII). Dissolved organic C (DOC) and dissolved organic N (DON) were determined using a TOC-VCPN analyzer (Shimadzu Scientific Instruments, Columbia). Soil pH was determined using a glass electrode in a 1:2.5 soil:water solution (w/v). Available phosphorus (AP) was extracted using NaHCO_3_ solution and was measured using the Mo-Sb colorimetric method. Available potassium (AK) was extracted with neutral ammonium acetate and was determined using flame photometry. The soil microbial biomass carbon (MBC) was determined using the chloroform fumigation extraction method^51^.

During each soil sampling period, macrofauna (e.g., Hymenoptera, Diptera, Orthoptera, Scolopendromorpha, Archaeogastropoda, Araneida, and Oligochaeta plesiopora) were collected by hand using a large sample corer (50 × 50 × 20 cm). Soil microarthropods were extracted from 100 g of soil (fresh weight) using modified Tullgren extractors^52^. All extracted soil faunal samples were preserved in 75% ethanol and subsequently sorted under a dissecting microscope (LeicaMZ 125, Germany) into broad taxonomic groups including Oribatids, Prostigmata, Mesostigmata, Collembola, Diptera larvae, Orthoptera, and Others (including Astigmata, Opiliones, Protura, Psocoptera, Blattaria, Symphyla, and the larvae of Lepidoptera, Hemiptera, Thysanoptera, Homoptera, and Dermaptera).

### Data analysis

We quantified the abundance and diversity soil faunal community using total abundance (TA), total groups (TG), Shannon-Wiener index (H′)^53^, and Margalef richness index (D)^54^, abundance of individual groups. We used analysis of variance (ANOVA) to test the effects of stand age, fertilization and their interaction term on abundance and diversity indices. When the effect was significant (α = 0.05), we used posthoc Tukey’s HSD test to examine the differences among stand age classes and fertilization treatments. We used Permutational Multivariate ANOVA (perMANOVA)^55^ to test the effects of stand age, fertilization and their interaction on fauna composition by including the abundances of individual groups as dependent variables.

To gain a mechanistic understanding of the changes in soil fauna, we tested how stand age and fertilization affected MBC, DOC, TN, DON, C:N, AP, and AK, and assessed the associations between these soil variables with the abundance and diversity of soil fauna by Pearson correlation analysis. We used principle component analysis (PCA) to determine the associations between the change in soil faunal community composition and soil variables.

It was necessary to transform the variables of soil faunal group and soil fertility by log(x + 1) to meet parametric assumptions of normality and homogeneity. All statistical analyses were performed using the SPSS version 21 software package (SPSS Inc., Chicago, IL, USA) or R version 2.10.1 with the VEGAN package (R Foundation for Statistical Computing).

## Additional Information

**How to cite this article**: Wang, S. *et al.* Fertilizer regime impacts on abundance and diversity of soil fauna across a poplar plantation chronosequence in coastal Eastern China. *Sci. Rep.* **6**, 20816; doi: 10.1038/srep20816 (2016).

## Figures and Tables

**Table 1:** The effects (F statistics) of stand age (A) and fertilization (F) on total abundance (ind.m^−2^), diversity, and individual group abundance of soil fauna.

Source	df	TA	TG	H′	D	O	P	M	C	Di	Or.	Others
A	2	9.89**	3.58	8.14*	7.58*	5.48*	8.28*	3.16	9.14**	7.25*	2.22	3.23
F	4	7.23*	4.34	7.62*	7.05*	6.29*	6.35*	3.02	9.26**	5.19*	2.76	2.65
A × F	8	6.16*	2.26	5.45*	5.79*	4.94*	5.11*	1.96	9.37*	4.84*	1.75	2.08

TA: Total abundance; TG: Total groups; H′: Shannon-Wiener index; D: Margalef index; O: Oribatids; P: Prostigmata; M: Mesostigmata; C: Collembola; Di: Diptera larvae; Or: Orthoptera; Other: other soil fauna except for Oribatids, Prostigmata, Mesostigmata, Collembola, Diptera larvae, and Orthoptera. Significant levels: **P < 0.01, *P < 0.05.

**Table 2:** Effects of stand age (A) and fertilization (F) on the community composition of soil fauna.

Source	df	SS	MS	F	P
A	2	1133.5	1042.3	9.8	0.001
F	4	957.8	903.6	7.4	0.035
A × F	8	605.7	558.8	3.2	0.055

Data were analyzed through permutational multivariate ANOVA (perMANOVA) analysis and the significance of each test was assessed with 9999 permutations.

**Table 3:** Effects of fertilizer regimes on the mean soil chemical parameters (Mean ± SE) from September 2012 to June 2013 across the three age classes.

Age (years)	Parameter	CK	OM1	OM2	NPK1	NPK2
4	MBC (g kg^−1^)	0.34 ± 0.12 ^c^	0.56 ± 0.16 ^b^	0.67 ± 0.21 ^a^	0.38 ± 0.12 ^c^	0.41 ± 0.13 ^c^
	DOC (mg kg^−1^)	204 ± 31.3 ^d^	266 ± 42.5 ^c^	354 ± 55.1 ^a^	221 ± 36.6 ^d^	304 ± 32.4 ^b^
	TN (g kg^−1^)	0.96 ± 0.18 ^c^	1.26 ± 0.22 ^b^	1.35 ± 0.31^a^	1.24 ± 0.13 ^b^	1.37 ± 0.33 ^a^
	DON (mg kg^−1^)	24.3 ± 2.31 ^d^	30.6 ± 2.43 ^c^	34.8 ± 3.11 ^b^	28.5 ± 2.68 ^c^	41.1 ± 2.87 ^a^
	C:N	14.6 ± 1.22 ^a^	12.1 ± 1.92 ^a^	12.1 ± 1.68 ^a^	11.6 ± 2.07 ^a^	11.3 ± 1.3 ^a^
	AP (g kg^−1^)	0.49 ± 0.17 ^d^	0.62 ± 0.17 ^c^	0.68 ± 0.22 ^c^	0.76 ± 0.21 ^b^	0.84 ± 0.32 ^a^
	AK (g kg^−1^)	6.34 ± 0.62 ^d^	7.66 ± 0.66 ^c^	8.45 ± 0.73 ^b^	8.68 ± 0.73 ^b^	9.72 ± 0.84 ^a^
9	MBC (g kg^−1^)	0.55 ± 0.14 ^c^	0.72 ± 0.14 ^b^	0.86 ± 0.14 ^a^	0.58 ± 0.14 ^c^	0.60 ± 0.14 ^c^
	DOC (mg kg^−1^)	235 ± 32.0 ^e^	295 ± 45.7 ^b^	331 ± 51.1 ^a^	279 ± 33.2 ^c^	255 ± 25.1 ^d^
	TN (g kg^−1^)	1.03 ± 0.09 ^e^	1.28 ± 0.18 ^d^	1.42 ± 0.26 ^b^	1.36 ± 0.22 ^c^	1.52 ± 0.32 ^a^
	DON (mg kg^−1^)	26.3 ± 2.25 ^e^	28.6 ± 2.72^d^	35.8 ± 3.38 ^b^	30.0 ± 2.21 ^c^	43.4 ± 2.54 ^a^
	C:N	14.2 ± 2.38 ^a^	12.5 ± 2.18 ^b^	11.4 ± 1.97 ^b^	11.4 ± 1.69 ^b^	10.6 ± 1.89 ^b^
	AP (g kg^−1^)	0.54 ± 0.22 ^d^	0.72 ± 0.22 ^c^	0.78 ± 0.22 ^c^	0.88 ± 0.22 ^b^	0.91 ± 0.22 ^a^
	AK (g kg^−1^)	6.48 ± 0.64 ^d^	7.79 ± 0.68 ^c^	8.62 ± 0.73 ^b^	8.65 ± 0.82 ^b^	9.84 ± 0.85 ^a^
20	MBC (g kg^−1^)	0.74 ± 0.16 ^c^	0.85 ± 0.15 ^b^	0.98 ± 0.15 ^a^	0.76 ± 0.15 ^c^	0.79 ± 0.15 ^c^
	DOC (mg kg^−1^)	283 ± 34.1 ^c^	372 ± 45.5 ^a^	322 ± 41.3 ^b^	300 ± 30.5 ^c^	335 ± 41.7 ^a^
	TN (g kg^−1^)	1.24 ± 0.16 ^c^	1.35 ± 0.10 ^b^	1.54 ± 0.25 ^a^	1.26 ± 0.14 ^c^	1.27 ± 0.11 ^c^
	DON (mg kg^−1^)	29.3 ± 2.42 ^c^	30.3 ± 2.53 ^b^	36.7 ± 3.03 ^a^	29.2 ± 2.85 ^c^	30.5 ± 3.15 ^c^
	C:N	14.3 ± 5.22 ^a^	14.5 ± 1.31 ^a^	11.8 ± 1.39 ^b^	14.1 ± 0.87 ^a^	14.9 ± 0.90 ^a^
	AP (g kg^−1^)	0.61 ± 0.24 ^d^	0.75 ± 0.24 ^c^	0.88 ± 0.24 ^b^	0.89 ± 0.24 ^b^	0.98 ± 0.24 ^a^
	AK (g kg^−1^)	7.33 ± 0.65 ^d^	8.54 ± 0.77 ^c^	9.56 ± 0.82 ^b^	9.67 ± 0.94 ^b^	10.43 ± 0.96 ^a^

Treatments with the same letter on the same date were not significantly different in each age class (ANOVA with Tukey-HSD, *p* < 0.05; *n* = 3). CK: unfertilized control; OM1: low level of organic matter fertilizer; OM2: high level of organic matter fertilizer; NPK1: low level of inorganic fertilizer; NPK2: high level of inorganic fertilizer; MBC: microbial biomass carbon; TN: total nitrogen; DOC: dissolved organic carbon; DON: dissolved organic nitrogen; C:N: carbon-nitrogen ratio; AP: available phosphorus; AK: available potassium.

**Table 4:** Correlations between individual group and diversity of soil fauna and soil chemical characteristics of the three age classes under the fertilizations.

Variables	MBC (g kg^−1^kg^−1^)	TN (g kg^−1^)	C:N	DOC (mg kg^−1^)	DON (mg kg^−1^)	AP (g kg^−1^)	AK (g kg^−1^)
Oribatids	0.94**	0.83	0.38	0.96**	0.77	0.88**	0.90*
Prostigmata	0.87*	0.87*	−0.21	0.88*	0.88*	0.86*	0.89*
Mesostigmata	0.85*	0.55	0.34	0.87*	−0.56	0.90*	0.89*
Collembola	0.93**	0.72	0.51	0.91**	0.79	0.85*	0.91**
Diptera larvae	0.63	0.65	−0.18	0.67	0.75	0.76	0.64
Orthoptera	0.52	0.25	0.47	0.46	0.40	0.53	0.58
TA	0.92**	0.75	0.85*	0.93**	0.92**	0.89**	0.91**
H′	0.91**	0.76	0.79	0.90*	0.91**	0.91**	0.89*
D	0.86*	0.61	0.81	0.85*	0.86*	0.88*	0.92**

Values are Pearson correlation coefficients; **P < 0.01,*P < 0.05. MBC: microbial biomass carbon; TN: total nitrogen; DOC: dissolved organic carbon; DON: dissolved organic nitrogen; C:N: carbon-nitrogen ratio; AP: available phosphorus; AK: available potassium; TA: Total abundance; H′: Shannon-Wiener index, and D: Margalef index.

**Table 5:** Stand characteristics along an age chronosequence in the coastal region of eastern China.

Age (years)	Stand density (trees ha^−1^)	Major understorey plants	Soil types	Height (m)	DBH (cm)	Canopy cover (%)	pH	Soil bulk density (g cm^−3^)	Total organic carbon (g kg^−1^)	Total organic nitrogen (g kg^−1^)
4	667	*Humulus scandens, Pteris biaurita*	Sandy loam soil	18.1 ± 1.8	16.2 ± 0.9	43	8.15 ± 0.26	1.13 ± 0.11	13.9 ± 0.47	0.91 ± 0.18
9	333	*Artemisia argyi, Cayratia albifolia, Convolvus arvensis*	Sandy loam soil	21.3 ± 2.5	23.1 ± 1.5	55	8.19 ± 0.47	1.24 ± 0.13	14.2 ± 0.52	0.98 ± 0.11
20	208	*Humulus scandens, Artemisia argyi, Pteris biaurita*	Sandy loam soil	26.6 ± 3.9	30.6 ± 1.8	75	8.24 ± 0.22	1.31 ± 0.15	14.3 ± 0.13	1.12 ± 0.16

Values of height, diameter at breast height (DBH), pH, soil bulk density, total organic carbon and nitrogen are mean  ±  SE (*n* = 3).
